# Supplementary material for: Endothelial MT1‐MMP targeting limits intussusceptive angiogenesis and colitis via TSP1/nitric oxide axis
Source: EMBO Mol Med. 2019 Dec 3;12(2):e10862. doi: 10.15252/emmm.201910862 (PMC7005619; doi:10.15252/emmm.201910862)
Supplement: Supplementary file 1 — Appendix [file EMMM-12-e10862-s001.pdf]

Appendix

Endothelial MT1-MMP targeting limits intussusceptive angiogenesis and colitis via TSP1-nitric oxide axis

Sergio Esteban, Cristina Clemente, Agnieszka Koziol, Pilar Gonzalo, Cristina Rius, Fernando Martínez, Pablo M. Linares, María Chaparro, Ana Urzainqui, Vicente Andrés, Motoharu Seiki, Javier P. Gisbert, and Alicia G. Arroyo

Appendix table of contents

Appendix Figure S1. MT1-MMP is expressed in endothelial cells of the mucosal plexus in the inflamed colon.

Appendix Figure S2. MT1-MMP deletion in endothelial cells reduces vascular leakage but does not influence myeloid cell infiltrate during early colitis.

Appendix Figure S3. Mouse aortic endothelial cells from MT1$^{i\Delta EC}$ mice produce lower amounts of nitric oxide.

Appendix Figure S4. Effects of MT1-MMP inhibition and of TSP1 on nitric oxide production by human endothelial cells.

Table S1. List of potential cleavage sites for MT1-MMP (MMP14) in thrombospondin-1 (TSP1).

Table S2. List of qPCR primers used for mouse (m) and human (h) genes in this study.

Table S3. List of exact p-values for graphs in Main Figures and Expanded View Figures.

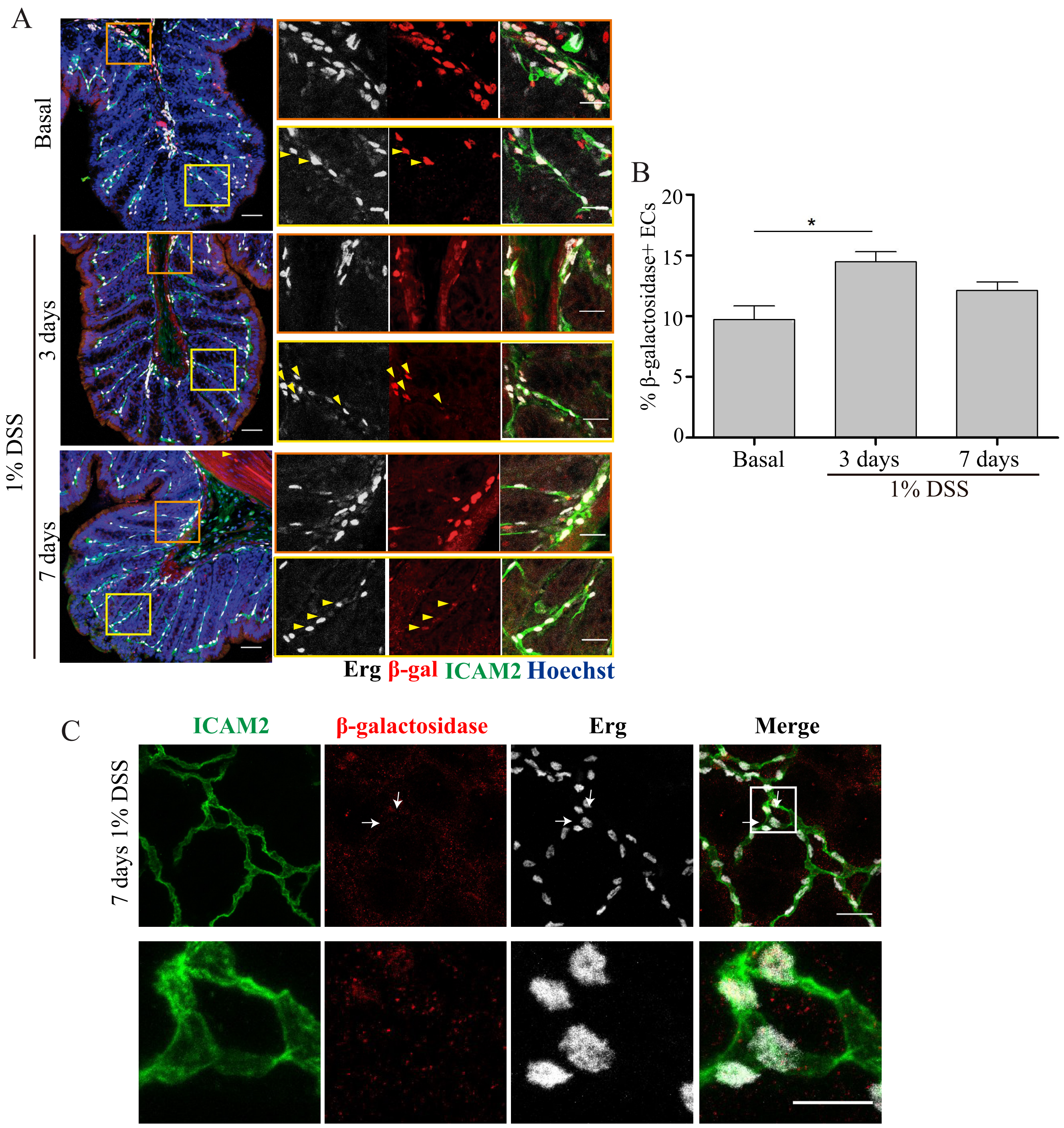

Appendix Figure S1. MT1-MMP is expressed in endothelial cells of the mucosal plexus in the inflamed colon. (**A**) Representative maximum intensity projection images of colon sections stained for ICAM2 (green), β-galactosidase (red), Erg (gray), and Hoechst (blue, nuclei) from $MT1^{lacZ/+}$ reporter mice left untreated (basal) or treated for 3 or 7 days with 1% DSS. Magnified regions including mucosal arterioles (orange box) or capillaries (yellow box) are shown to the right. Arrowheads indicate β-gal-positive ECs in the capillaries. Scale bar, 50 µm (left panels) and 25 µm (magnified views). (**B**) Bar graph shows the percentage of endothelial cells (Erg) positive for β-galactosidase; n=3-5 mice per condition. Data are shown as mean ± SEM and were tested by Student t-test; *$p<0.05$. (**C**) Representative maximum intensity projection images of staining for ICAM2 (green), β-galactosidase (red), and Erg (gray) in whole-mount distal colon mucosal plexus from $MT1^{lacZ/+}$ reporter mice treated for 7 days with 1% DSS. Scale bar, 20 µm. White arrows indicate patches of β-galactosidase-positive endothelial cells.

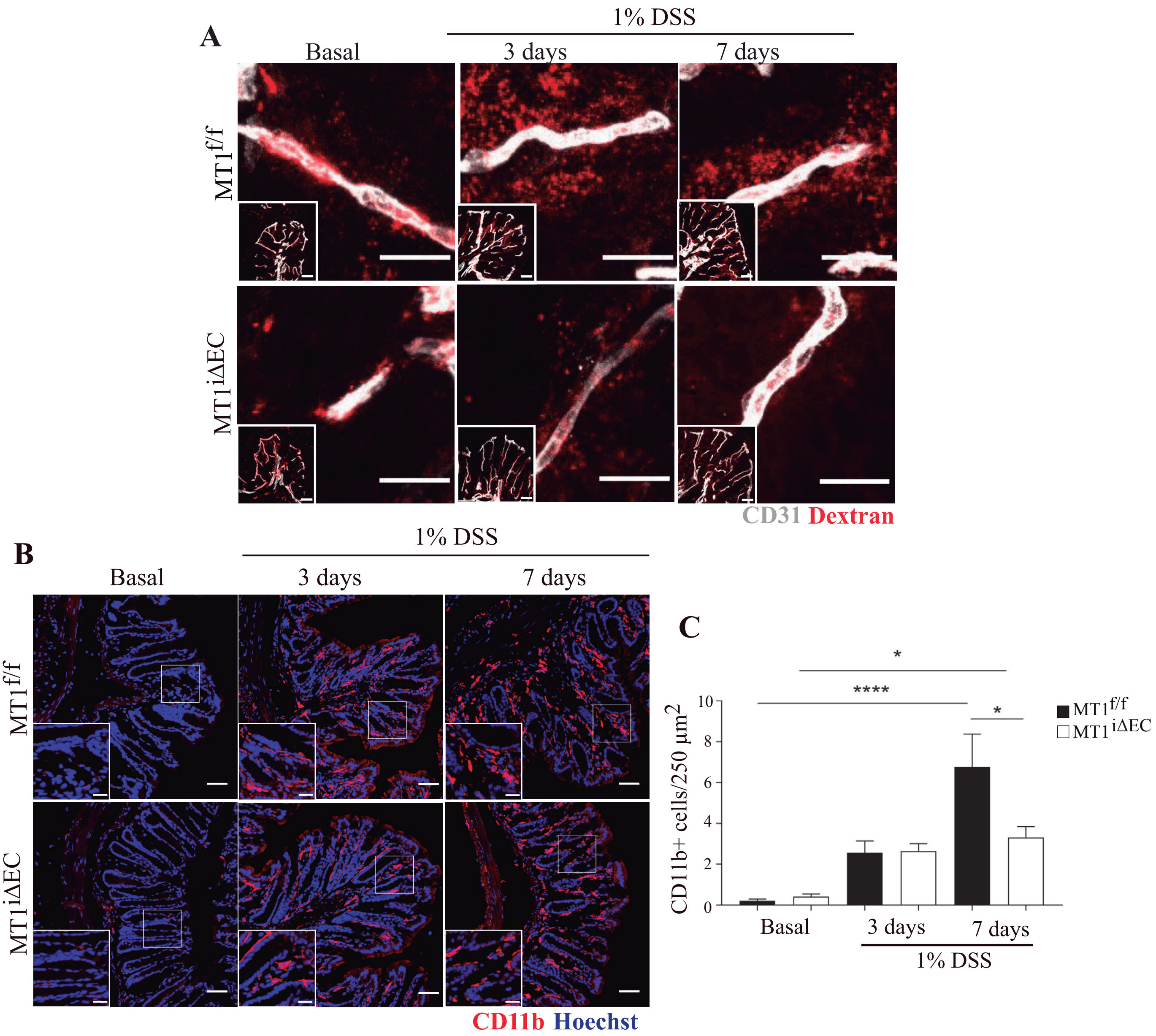

Appendix Figure S2. MT1-MMP deletion in endothelial cells reduces vascular leakage but does not influence myeloid cell infiltrate during early colitis. (**A**) Representative maximum intensity projection images of whole-mount distal colons stained for CD31 (gray) and 70 KD Dextran (red) in $MT1^{f/f}$ and $MT1^{i\Delta EC}$ mice left untreated (basal) or treated with 1% DSS for 3 or 7 days. Scale bar, 50 µm and 20 µm in the magnified views. (**B**) Representative maximum intensity projection images of staining for CD11b (red) and Hoechst (blue, nuclei) in colon sections from $MT1^{f/f}$ and $MT1^{i\Delta EC}$ mice left untreated (basal) or treated with 1% DSS for 3 or 7 days. Magnified views are shown in the insets. Scale bar, 50 µm and 25 µm in the magnified. (**C**) Quantification of CD11b+ cells in sections as in A; n=4-5 mice per genotype and time point. Data are shown as mean ± SEM and were tested by one-way ANOVA with Benjamini and Hochbers post-test; $*p<0.05$, $****p<0.0001$.

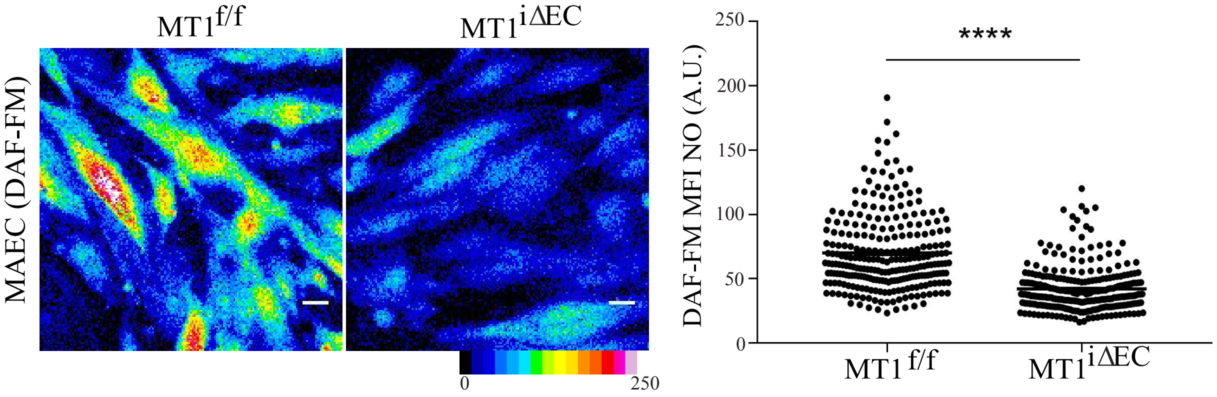

Appendix Figure S3. Mouse aortic endothelial cells from MT1i$^{\Delta EC}$ mice produce lower amounts of nitric oxide. Representative images are shown of mouse aortic endothelial cells from MT1$^{f/f}$ and MT1$^{i\Delta EC}$ mice loaded with the DAF-FM nitric oxide probe (pseudocolor representation of green channel intensity); scale bar, 25 μm. The graph shows DAF-FM mean fluorescence intensity (MFI) in n= 263-266 cells analyzed per genotype in 3 independent experiments. Data are shown as individual cell values and the means and were analyzed by Mann-Whitney test; ****$p<0.0001$

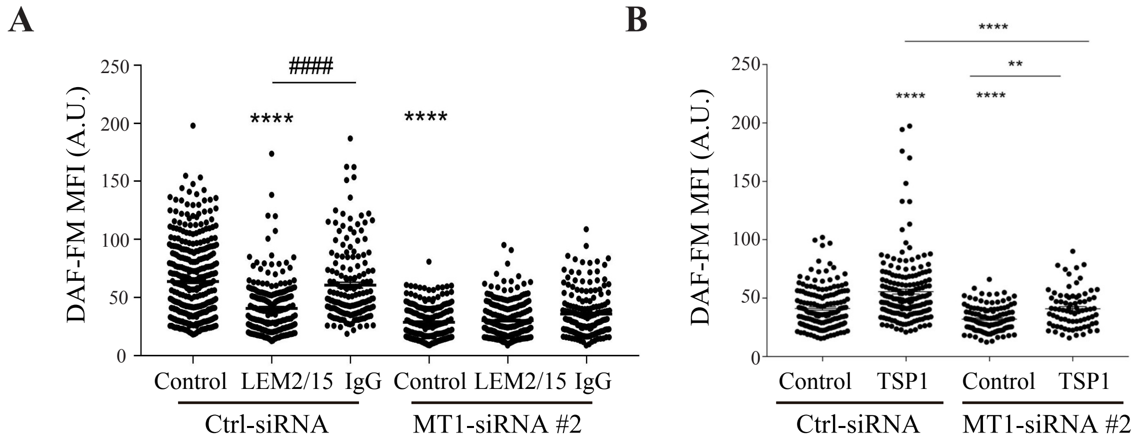

Appendix Figure S4. Effects of MT1-MMP inhibition and of TSP1 on nitric oxide production by human endothelial cells. (**A**) DAF-FM mean fluorescence intensity (MFI) in HUVEC expressing control or MT1-MMP siRNA and left untreated (Control) or treated with the inhibitory anti-MT1-MMP antibody LEM-2/15 (10 µg/ml for 24 h) or its corresponding IgG1 isotype control (IgG); n=128-360 cells analyzed per condition in 3 independent experiments. (**B**) DAF-FM mean fluorescence intensity (MFI) values in HUVEC expressing control or MT1-MMP siRNA cultured on plates coated with 1% gelatin or with 1% gelatin plus 200 ng/ml TSP1; n= 78-184 cells in 3 independent experiments. Single cell values and the means are shown. Data were tested by Mann-Whitney test **$p<0.01$ #### or ****$p<0.0001$. * mark comparison with Ctrl-siRNA; # mark comparison with isotype IgG.

P1 position	Residues	PWM ^ Score	Sec Struct pred	Disorder	Transmemb domain	N-mass	C-mass
6	GLAWG-LGVLF	3.71	____HHHEEE	....	OOOOOOOOOO	633.28	128683.54
11	LGVLF-LMHVC	5.27	HHEEEEEEE_	....	OOOOOOOOOO	1162.60	128154.22
93	LLLAS-LRQMK	6.64	HHHHHHHHHH	....	OOOOOOOOOO	9933.05	119383.77
193	RDLAS-IARLR	1.17	HHHHHHHHHH	....	OOOOOOOOOO	21025.78	108291.04
225	TTPED-ILRNK	3.00	_____EE___	....	OOOOOOOOOO	24538.63	104778.19
253	GSSPA-IRTNY	0.54	_____EEEE_	....	OOOOOOOOOO	27366.04	101950.78
286	LELRG-LRTIV	7.90	HHHH___EEE	....	OOOOOOOOOO	31010.84	98305.98
440	GGWSH-WSPWS	1.33	__________	....	OOOOOOOOOO	48377.51	80939.31
467	NSPSP-QMNGK	1.11	__________	**********	OOOOOOOOOO	51266.82	78050.00
578	ACPPG-YSGNG	0.89	__________	....	OOOOOOOOOO	62958.91	66357.91
668	NYLGH-YSDPM	1.56	__________	....	OOOOOOOOOO	72791.09	56525.73
1024	DDYAG-FVFGY	0.75	____EEEEEE	....	OOOOOOOOOO	112343.48	16973.34
1060	QGYSG-LSVKV	4.42	_____EEEEE	....	OOOOOOOOOO	116595.46	12721.36
1075	GPGEH-LRNAL	1.85	_____EEEEE	....	OOOOOOOOOO	118101.23	11215.59
1092	GQVRT-LWHDP	1.76	_EEEEEE___	....	OOOOOOOOOO	120003.22	9313.60
1099	HDPRH-IGWKD	5.66	_________H	....	OOOOOOOOOO	120944.68	8372.14

Appendix Table S1. **List of potential cleavage sites for MT1-MMP (MMP14) in thrombospondin-1 (TSP1)**. Predicted sites for human MT1-MMP-mediated cleavage in TSP1 are shown with their cleavage scores (http://cleavpredict.sanfordburnham.org/). Candidate sites were filtered by *in silico* modeling and scored according to the peptide cleavage matrix in the MEROPS database (http://merops.sanger.ac.uk/). The selected cleavage sites in TSP1 are highlighted in yellow.

Gene	Direction	Primer Sequence
mMmp14	Forward	5´- CAGTATGGCTACCTACCTCCAG-3
	Reverse	5´- GCCTTGCCTGTCACTTGTAAA -3´
mCdh5	Forward	5´-AAAGAACTGGACAGAGAAA-3´
	Reverse	5´- GGTTGTCATTCTCATCCAAG -3
mTbp	Forward	5´- GCTCTGGAATTGTACCGCAG-3´
	Reverse	5´- CTGGCTCATAGCTCTTGGCTC-3´
mGapdh	Forward	5´- AATGCATCCTGCACCACCAA-3´
	Reverse	5´- GTGGCAGTGATGGCATGGAC-3´
hNOS3	Forward	5´-GGCTGGGTTTAGGGCTGTG-3´
	Reverse	5´-TGAGGGTGTCGTAGGTGATG-3´
hMMP14	Forward	5´-GGCTACAGCAATATGGCTACC-3´
	Reverse	5´-GATGGCCGCTGAGAGTGAC-3´
hGAPDH	Forward	5´-GGAGCGAGATCCCTCCAAAAT-3´
	Reverse	5´-GGCTGTTGTCATACTTCTCATGG-3´
hHPRT	Forward	5´-CCTGGCGTCGTGATTAGTGAT-3´
	Reverse	5´-AGACGTTCAGTCCTGTCCATAA-3´
hYWHAB	Forward	5´-CCTGCATGAAGTCTGTAACTGAG-3´
	Reverse	5´-GACCTACGGGCTCCTACAACA-3´

Appendix Table S2. **List of qPCR primers used for mouse (m) and human (h) genes in this study**.

	Comparison	p-value
Figure 1B	CN MT1f/f vs. CN MT1EC	0,861
	CN MT1f/f vs. 3d 1DSS MT1f/f	0,0057
	CN MT1f/f vs. 3d 1DSS MT1EC	0,6013
	CN MT1f/f vs. 7d 1DSS MT1f/f	0,066
	CN MT1f/f vs. 7d 1DSS MT1EC	0,9492
	CN MT1EC vs. 3d 1DSS MT1f/f	0,0028
	CN MT1EC vs. 3d 1DSS MT1EC	0,4774
	CN MT1EC vs. 7d 1DSS MT1f/f	0,038
	CN MT1EC vs. 7d 1DSS MT1EC	0,7927
	3d 1DSS MT1f/f vs. 3d 1DSS MT1EC	0,0225
	3d 1DSS MT1f/f vs. 7d 1DSS MT1f/f	0,2181
	3d 1DSS MT1f/f vs. 7d 1DSS MT1EC	0,0026
	3d 1DSS MT1EC vs. 7d 1DSS MT1f/f	0,198
	3d 1DSS MT1EC vs. 7d 1DSS MT1EC	0,6026
	7d 1DSS MT1f/f vs. 7d 1DSS MT1EC	0,0426
	Comparison	p-value
Figure 1C	MT1f/f CN vs. MT1ΔEC CN	0,7134
	MT1f/f CN vs. MT1f/f DSS	0,0013
	MT1f/f CN vs. MT1ΔEC DSS	0,4721
	MT1ΔEC CN vs. MT1f/f DSS	0,0005
	MT1ΔEC CN vs. MT1ΔEC DSS	0,2821
	MT1f/f DSS vs. MT1ΔEC DSS	0,007
	Comparison	p-value
Figure 2B	cre- 3dias vs. cre + 3 dias	0,0937
	cre- 3dias vs. cre-7dias	>0,9999
	cre- 3dias vs. cre+7 dias	>0,9999
	cre + 3 dias vs. cre-7dias	0,3824
	cre + 3 dias vs. cre+7 dias	0,3775
	cre-7dias vs. cre+7 dias	0,0032
	Comparison	p-value
Figure 2E	1	
	CONTROL CRE- vs. CONTROL CRE+	0,8605
	CONTROL CRE- vs. 1% DSS CRE-	0,9948
	CONTROL CRE- vs. 1% DSS CRE+	0,6912
	CONTROL CRE+ vs. 1% DSS CRE-	0,8501
	CONTROL CRE+ vs. 1% DSS CRE+	0,5559
	1% DSS CRE- vs. 1% DSS CRE+	0,6845
	2	
	CONTROL CRE- vs. CONTROL CRE+	0,8111
	CONTROL CRE- vs. 1% DSS CRE-	0,9635
	CONTROL CRE- vs. 1% DSS CRE+	0,6467
	CONTROL CRE+ vs. 1% DSS CRE-	0,8403
	CONTROL CRE+ vs. 1% DSS CRE+	0,8322
	1% DSS CRE- vs. 1% DSS CRE+	0,6685
	3	
	CONTROL CRE- vs. CONTROL CRE+	0,6478

	CONTROL CRE- vs. 1% DSS CRE-	0,8754
	CONTROL CRE- vs. 1% DSS CRE+	0,8069
	CONTROL CRE+ vs. 1% DSS CRE-	0,7529
	CONTROL CRE+ vs. 1% DSS CRE+	0,8138
	1% DSS CRE- vs. 1% DSS CRE+	0,9299
	4	
	CONTROL CRE- vs. CONTROL CRE+	0,9049
	CONTROL CRE- vs. 1% DSS CRE-	0,4698
	CONTROL CRE- vs. 1% DSS CRE+	0,7689
	CONTROL CRE+ vs. 1% DSS CRE-	0,3917
	CONTROL CRE+ vs. 1% DSS CRE+	0,6713
	1% DSS CRE- vs. 1% DSS CRE+	0,6445
	5	
	CONTROL CRE- vs. CONTROL CRE+	0,7488
	CONTROL CRE- vs. 1% DSS CRE-	0,0069
	CONTROL CRE- vs. 1% DSS CRE+	0,1306
	CONTROL CRE+ vs. 1% DSS CRE-	0,0022
	CONTROL CRE+ vs. 1% DSS CRE+	0,0614
	1% DSS CRE- vs. 1% DSS CRE+	0,1934
	6	
	CONTROL CRE- vs. CONTROL CRE+	0,9829
	CONTROL CRE- vs. 1% DSS CRE-	<0,0001
	CONTROL CRE- vs. 1% DSS CRE+	<0,0001
	CONTROL CRE+ vs. 1% DSS CRE-	<0,0001
	CONTROL CRE+ vs. 1% DSS CRE+	<0,0001
	1% DSS CRE- vs. 1% DSS CRE+	<0,0001
	7	
	CONTROL CRE- vs. CONTROL CRE+	0,972
	CONTROL CRE- vs. 1% DSS CRE-	<0,0001
	CONTROL CRE- vs. 1% DSS CRE+	<0,0001
	CONTROL CRE+ vs. 1% DSS CRE-	<0,0001
	CONTROL CRE+ vs. 1% DSS CRE+	<0,0001
	1% DSS CRE- vs. 1% DSS CRE+	<0,0001
	Comparison	p-value
Figure 3C	CRE- vs. CRE+	0,1624
	Comparison	p-value
Figure 3D	CRE- vs. CRE+	0,00124

	Comparison	p-value
Figure 3F	Cre- vs. Cre - ACH	0,0053
	Cre- vs. Cre - ACH no	0,0026
	Cre + vs. Cre + ACH	0,5619
	Cre + vs. Cre + ACH no	<0,0001
	Cre - ACH vs. Cre + ACH	0,02
	Cre - ACH no vs. Cre + ACH no	0,1083

	Cre- vs. Cre +	0,7287
	Cre - ACH vs. Cre - ACH no	0,5299
	Cre + ACH vs. Cre + ACH no	0,0001
	Comparison	p-value
Figure 4A	control vs. siRNA 77	< 0,0001
	Comparison	p-value
Figure 4C	control vs. siRNA 77 (eNOS)	0,0001
	control vs. siRNA 79 (eNOS)	0,0001
	control vs. siRNA 77 (MT1-MMP)	0,0001
	control vs. siRNA 79 (MT1-MMP)	0,0001
	Comparison	p-value
Figure 4D	c vs. 77 (eNOS)	0,00151
	c vs. 79 (eNOS)	0,0034
	c vs. 77 (MT1-MMP)	0,0003
	c vs. 79 (MT1-MMP)	0,0003
	Comparison	p-value
Figure 5	cre- vs. cre- dss	0,0001
	cre- vs. cre+	0,622
	cre- vs. cre + dss	0,2252
	cre- vs. FL	<0,0001
	cre- vs. EA	0,0246
	cre- vs. YF	0,0002
	cre- dss vs. cre+	0,0018
	cre- dss vs. cre + dss	0,011
	cre- dss vs. FL	0,9979
	cre- dss vs. EA	0,0534
	cre- dss vs. YF	0,7293
	cre+ vs. cre + dss	0,5112
	cre+ vs. FL	0,0013
	cre+ vs. EA	0,1212
	cre+ vs. YF	0,0032
	cre + dss vs. FL	0,0087
	cre + dss vs. EA	0,3904
	cre + dss vs. YF	0,0199
	FL vs. EA	0,0445
	FL vs. YF	0,7166
	EA vs. YF	0,095
	Comparison	p-value
Figure 6C	CRE- vs. CRE+	0,0254
	Comparison	p-value
Figure 7B	gel wt vs. av wt	<0,0001
	gel wt vs. cd47 wt	<0,0001
	gel wt vs. igg1 wt	>0,9999
	gel wt vs. IGG1 10 wt	>0,9999
	gel wt vs. gel sirna	<0,0001
	gel wt vs. av sirna	<0,0001
	gel wt vs. cd47 sirna	<0,0001
	gel wt vs. igg1 sirna	<0,0001
	gel wt vs. IGG1 10 sirna	<0,0001
	av wt vs. cd47 wt	0,4821

av wt vs. igg1 wt	<0,0001
av wt vs. IGG1 10 wt	<0,0001
av wt vs. gel sirna	0,0588
av wt vs. av sirna	>0,9999
av wt vs. cd47 sirna	0,4926
av wt vs. igg1 sirna	>0,9999
av wt vs. IGG1 10 sirna	>0,9999
cd47 wt vs. igg1 wt	<0,0001
cd47 wt vs. IGG1 10 wt	<0,0001
cd47 wt vs. gel sirna	<0,0001
cd47 wt vs. av sirna	0,7843
cd47 wt vs. cd47 sirna	<0,0001
cd47 wt vs. igg1 sirna	>0,9999
cd47 wt vs. IGG1 10 sirna	>0,9999
igg1 wt vs. IGG1 10 wt	>0,9999
igg1 wt vs. gel sirna	<0,0001
igg1 wt vs. av sirna	<0,0001
igg1 wt vs. cd47 sirna	<0,0001
igg1 wt vs. igg1 sirna	<0,0001
igg1 wt vs. IGG1 10 sirna	<0,0001
IGG1 10 wt vs. gel sirna	<0,0001
IGG1 10 wt vs. av sirna	<0,0001
IGG1 10 wt vs. cd47 sirna	<0,0001
IGG1 10 wt vs. igg1 sirna	<0,0001
IGG1 10 wt vs. IGG1 10 sirna	<0,0001
gel sirna vs. av sirna	0,1306
gel sirna vs. cd47 sirna	>0,9999
gel sirna vs. igg1 sirna	0,1316
gel sirna vs. IGG1 10 sirna	0,0886
av sirna vs. cd47 sirna	0,7833
av sirna vs. igg1 sirna	>0,9999
av sirna vs. IGG1 10 sirna	>0,9999
cd47 sirna vs. igg1 sirna	0,72
cd47 sirna vs. IGG1 10 sirna	0,5692
igg1 sirna vs. IGG1 10 sirna	>0,9999

Figure 7C

Comparison	p-value
GELATINA WT vs. RGD 200 WT	<0,0001
GELATINA WT vs. RGD 20WT	<0,0001
GELATINA WT vs. RGD 2nM WT	<0,0001
GELATINA WT vs. RDAS 200 wt	>0,9999
GELATINA WT vs. RDAS20 wt	>0,9999
GELATINA WT vs. RDAS 2n wt	>0,9999
GELATINA WT vs. GELATINA sirna	<0,0001
GELATINA WT vs. RGD 200 sirna	<0,0001
GELATINA WT vs. RGD 20 sirna	<0,0001
GELATINA WT vs. RGD 2nM sirna	<0,0001
GELATINA WT vs. RDAS 200 sirna	<0,0001
GELATINA WT vs. RDAS20 sirna	<0,0001
GELATINA WT vs. RDAS 2 sirna	<0,0001
RGD 200 WT vs. RGD 20WT	>0,9999

RGD 200 WT vs. RGD 2nM WT	>0,9999
RGD 200 WT vs. RDAS 200 wt	0,0001
RGD 200 WT vs. RDAS20 wt	<0,0001
RGD 200 WT vs. RDAS 2n wt	<0,0001
RGD 200 WT vs. GELATINA sirna	<0,0001
RGD 200 WT vs. RGD 200 sirna	0,0417
RGD 200 WT vs. RGD 20 sirna	0,0015
RGD 200 WT vs. RGD 2nM sirna	<0,0001
RGD 200 WT vs. RDAS 200 sirna	0,0622
RGD 200 WT vs. RDAS20 sirna	<0,0001
RGD 200 WT vs. RDAS 2 sirna	0,037
RGD 20WT vs. RGD 2nM WT	>0,9999
RGD 20WT vs. RDAS 200 wt	<0,0001
RGD 20WT vs. RDAS20 wt	<0,0001
RGD 20WT vs. RDAS 2n wt	<0,0001
RGD 20WT vs. GELATINA sirna	<0,0001
RGD 20WT vs. RGD 200 sirna	0,5329
RGD 20WT vs. RGD 20 sirna	0,0318
RGD 20WT vs. RGD 2nM sirna	0,0001
RGD 20WT vs. RDAS 200 sirna	0,7301
RGD 20WT vs. RDAS20 sirna	0,0006
RGD 20WT vs. RDAS 2 sirna	0,5109
RGD 2nM WT vs. RDAS 200 wt	<0,0001
RGD 2nM WT vs. RDAS20 wt	<0,0001
RGD 2nM WT vs. RDAS 2n wt	<0,0001
RGD 2nM WT vs. GELATINA sirna	<0,0001
RGD 2nM WT vs. RGD 200 sirna	0,0944
RGD 2nM WT vs. RGD 20 sirna	0,004
RGD 2nM WT vs. RGD 2nM sirna	<0,0001
RGD 2nM WT vs. RDAS 200 sirna	0,1373
RGD 2nM WT vs. RDAS20 sirna	<0,0001
RGD 2nM WT vs. RDAS 2 sirna	0,086
RDAS 200 wt vs. RDAS20 wt	>0,9999
RDAS 200 wt vs. RDAS 2n wt	0,3394
RDAS 200 wt vs. GELATINA sirna	<0,0001
RDAS 200 wt vs. RGD 200 sirna	<0,0001
RDAS 200 wt vs. RGD 20 sirna	<0,0001
RDAS 200 wt vs. RGD 2nM sirna	<0,0001
RDAS 200 wt vs. RDAS 200 sirna	<0,0001
RDAS 200 wt vs. RDAS20 sirna	<0,0001
RDAS 200 wt vs. RDAS 2 sirna	<0,0001
RDAS20 wt vs. RDAS 2n wt	>0,9999
RDAS20 wt vs. GELATINA sirna	<0,0001
RDAS20 wt vs. RGD 200 sirna	<0,0001
RDAS20 wt vs. RGD 20 sirna	<0,0001
RDAS20 wt vs. RGD 2nM sirna	<0,0001
RDAS20 wt vs. RDAS 200 sirna	<0,0001
RDAS20 wt vs. RDAS20 sirna	<0,0001
RDAS20 wt vs. RDAS 2 sirna	<0,0001
RDAS 2n wt vs. GELATINA sirna	<0,0001

	RDAS 2n wt vs. RGD 200 sirna	<0,0001
	RDAS 2n wt vs. RGD 20 sirna	<0,0001
	RDAS 2n wt vs. RGD 2nM sirna	<0,0001
	RDAS 2n wt vs. RDAS 200 sirna	<0,0001
	RDAS 2n wt vs. RDAS20 sirna	<0,0001
	RDAS 2n wt vs. RDAS 2 sirna	<0,0001
	GELATINA sirna vs. RGD 200 sirna	0,3107
	GELATINA sirna vs. RGD 20 sirna	>0,9999
	GELATINA sirna vs. RGD 2nM sirna	>0,9999
	GELATINA sirna vs. RDAS 200 sirna	0,2163
	GELATINA sirna vs. RDAS20 sirna	>0,9999
	GELATINA sirna vs. RDAS 2 sirna	0,233
	RGD 200 sirna vs. RGD 20 sirna	>0,9999
	RGD 200 sirna vs. RGD 2nM sirna	>0,9999
	RGD 200 sirna vs. RDAS 200 sirna	>0,9999
	RGD 200 sirna vs. RDAS20 sirna	>0,9999
	RGD 200 sirna vs. RDAS 2 sirna	>0,9999
	RGD 20 sirna vs. RGD 2nM sirna	>0,9999
	RGD 20 sirna vs. RDAS 200 sirna	>0,9999
	RGD 20 sirna vs. RDAS20 sirna	>0,9999
	RGD 20 sirna vs. RDAS 2 sirna	>0,9999
	RGD 2nM sirna vs. RDAS 200 sirna	>0,9999
	RGD 2nM sirna vs. RDAS20 sirna	>0,9999
	RGD 2nM sirna vs. RDAS 2 sirna	>0,9999
	RDAS 200 sirna vs. RDAS20 sirna	>0,9999
	RDAS 200 sirna vs. RDAS 2 sirna	>0,9999
	RDAS20 sirna vs. RDAS 2 sirna	>0,9999
	Comparison	p-value
Figure 7D	gel wt vs. cilen 200 wt	<0,0001
	gel wt vs. cilen 1 wt	<0,0001
	gel wt vs. gel sirna	<0,0001
	gel wt vs. cilen 200 sirna	<0,0001
	gel wt vs. cilen 1 sirna	<0,0001
	cilen 200 wt vs. cilen 1 wt	>0,9999
	cilen 200 wt vs. gel sirna	0,0157
	cilen 200 wt vs. cilen 200 sirna	<0,0001
	cilen 200 wt vs. cilen 1 sirna	<0,0001
	cilen 1 wt vs. gel sirna	<0,0001
	cilen 1 wt vs. cilen 200 sirna	<0,0001
	cilen 1 wt vs. cilen 1 sirna	<0,0001
	gel sirna vs. cilen 200 sirna	>0,9999
	gel sirna vs. cilen 1 sirna	0,7646
	cilen 200 sirna vs. cilen 1 sirna	>0,9999
	Comparison	p-value
Figure 7E	gel wt vs. nonamero 200	<0,0001
	gel wt vs. nonamero 2	<0,0001
	gel wt vs. Control 200	0,2515
	gel wt vs. control2	>0,9999
	gel wt vs. gel sirna	<0,0001
	gel wt vs. nonamero 200 sirna	<0,0001

	gel wt vs. nonamero 2 sirna	<0,0001
	gel wt vs. Control 200 sirna	<0,0001
	gel wt vs. control2 sirna	<0,0001
	nonamero 200 vs. nonamero 2	>0,9999
	nonamero 200 vs. Control 200	<0,0001
	nonamero 200 vs. control2	<0,0001
	nonamero 200 vs. gel sirna	>0,9999
	nonamero 200 vs. nonamero 200 sirna	>0,9999
	nonamero 200 vs. nonamero 2 sirna	>0,9999
	nonamero 200 vs. Control 200 sirna	>0,9999
	nonamero 200 vs. control2 sirna	>0,9999
	nonamero 2 vs. Control 200	<0,0001
	nonamero 2 vs. control2	<0,0001
	nonamero 2 vs. gel sirna	>0,9999
	nonamero 2 vs. nonamero 200 sirna	>0,9999
	nonamero 2 vs. nonamero 2 sirna	>0,9999
	nonamero 2 vs. Control 200 sirna	>0,9999
	nonamero 2 vs. control2 sirna	>0,9999
	Control 200 vs. control2	>0,9999
	Control 200 vs. gel sirna	<0,0001
	Control 200 vs. nonamero 200 sirna	<0,0001
	Control 200 vs. nonamero 2 sirna	<0,0001
	Control 200 vs. Control 200 sirna	<0,0001
	Control 200 vs. control2 sirna	<0,0001
	control2 vs. gel sirna	<0,0001
	control2 vs. nonamero 200 sirna	<0,0001
	control2 vs. nonamero 2 sirna	<0,0001
	control2 vs. Control 200 sirna	<0,0001
	control2 vs. control2 sirna	<0,0001
	gel sirna vs. nonamero 200 sirna	>0,9999
	gel sirna vs. nonamero 2 sirna	0,1851
	gel sirna vs. Control 200 sirna	>0,9999
	gel sirna vs. control2 sirna	0,0642
	nonamero 200 sirna vs. nonamero 2 sirna	>0,9999
	nonamero 200 sirna vs. Control 200 sirna	>0,9999
	nonamero 200 sirna vs. control2 sirna	>0,9999
	nonamero 2 sirna vs. Control 200 sirna	>0,9999
	nonamero 2 sirna vs. control2 sirna	>0,9999
	Control 200 sirna vs. control2 sirna	>0,9999
	Comparison	p-value
Figure 8B	CONTROL vs UC low activity	0,0003
	CONTROL vs UC high activity	0,9854
	UC low activity vs UC high activity	0,0003
	CONTROL vs CD low activity	0,0546
	CONTROL vs CD high activity	0,9472
	CD low activity vs CD high activity	0,0646
	Comparison	p-value
Figure 8D	IgG vs. LEM-2/15	0,0071
	Comparison	p-value
Figure 8G	GDGRADACK vs. GDGRGDACK	0,0438

	Comparison	p-value
EV3B	MT1f/f - MT1f/fVeCdhCreERT2	
	1	>0,9999
	2	0,9057
	3	>0,9999
	4	0,8801
	5	0,7547
	6	0,2915
	7	0,9657
	8	0,1929
	9	0,3272
	10	0,0745
	11	0,219
	12	<0,0001
	13	0,0041
	14	0,0008
	15	0,0022
	Comparison	p-value
EV 3C	MT1f/f - MT1f/fVeCdhCreERT2	
	1	>0,9999
	2	0,7699
	3	0,478
	4	0,7678
	5	0,7044
	6	0,9618
	7	0,8615
	8	0,6491
	9	0,5795
	10	0,1348
	11	0,0559
	12	0,0021
	13	0,0014
	14	0,0017
	15	0,004
	Comparison	p-value
EV 4D	MT1f/f - MT1f/fVeCdhCreERT2	0,0231
	Comparison	p-value
EV 5E	MT1 siRNA_Control vs MT1 siRNA_TSP1 full 200	0,3981
	MT1 siRNA_Control vs MT1 siRNA_E123CaG 200	0,021
	MT1 siRNA_TSP1 full 200 vs MT1 siRNA_E123CaG 200	0,0003
	Comparison	p-value
EV 5G	TSP1 full vs E123CaG 200	0,0444

Table S3. List of exact p-values for graphs in Main Figures and Expanded View Figures.
