# Supplementary material for: Endothelial MT1‐MMP targeting limits intussusceptive angiogenesis and colitis via TSP1/nitric oxide axis
Source: EMBO Mol Med. 2019 Dec 3;12(2):e10862. doi: 10.15252/emmm.201910862 (PMC7005619; doi:10.15252/emmm.201910862)
Supplement: Supplementary file 2 — Expanded View Figures PDF [file EMMM-12-e10862-s002.pdf]

Expanded View Figures

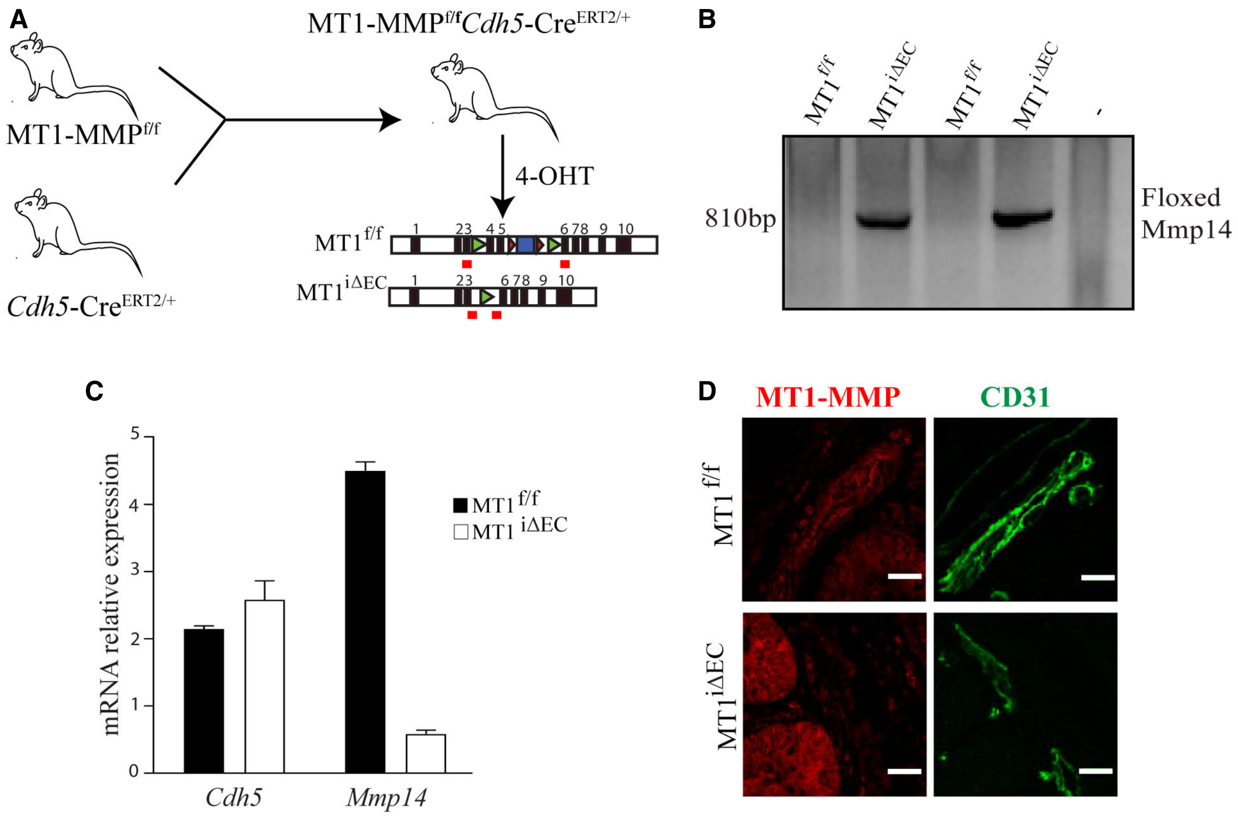

Figure EV1. Strategy and validation of endothelial cell-type-specific deletion of MT1-MMP in mice.

A Strategy for obtaining endothelial cell-specific MT1-MMP (*Mmp14*)-null mice by crossing MT1-MMP$^{f/f}$ mice (Gutierrez-Fernandez *et al*, 2015) with *Cdh5*-CreERT2/+ mice (Wang *et al*, 2010). The floxed and targeted Mmp14 alleles are illustrated.

B PCR of lung DNA from MT1$^{f/f}$ and MT1$^{i\Delta EC}$ mice detecting the targeted (floxed-out) Mmp14 allele (810 bp).

C qPCR analysis of relative *Mmp14* and *Cdh5* mRNA levels in sorted lung endothelial cells from MT1$^{f/f}$ and MT1$^{i\Delta EC}$ mice. n = 3 mice per genotype. Data are shown as mean ± SEM.

D Representative maximum-intensity projection images of staining for MT1-MMP (red) and CD31 (green) in colon sections from MT1$^{f/f}$ and MT1$^{i\Delta EC}$ mice. Scale bar, 10 μm.

Data information: Please see Appendix Table S3 for exact *P*-values.
Source data are available online for this figure.

© 2019 The Authors

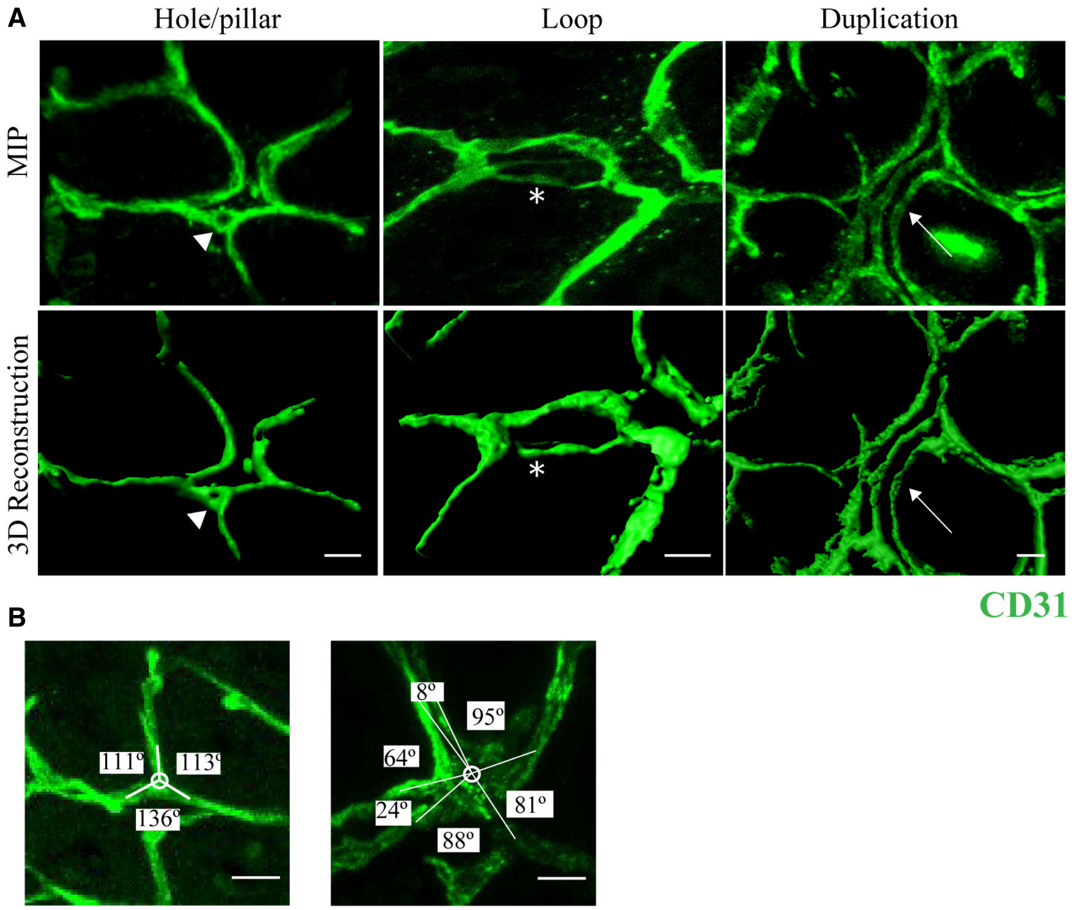

Figure EV2. Visualization of intussusceptive angiogenesis hallmarks in mouse colitis by confocal microscopy and 3D-image rendering.

A Representative maximum-intensity projection (MIP) images of mucosal plexus in whole-mount distal colon stained for CD31 (green) from a $MT1^{f/f}$ mouse treated with 1% DSS for 3 days (upper row). 3D rendering of the raw images was performed with Imaris® (lower panels). Holes, loops, and duplications are visible and indicated by arrowhead, asterisk, and arrow, respectively. Scale bar, 10 µm.

B Magnified view of a tri-corner in the colon mucosal plexus (left) and a splitting corner after 3 days of 1% DSS (right), illustrating quantification of the intercapillary angles (Ackermann *et al*, 2013). Scale bar, 10 µm.

 © 2019 The Authors

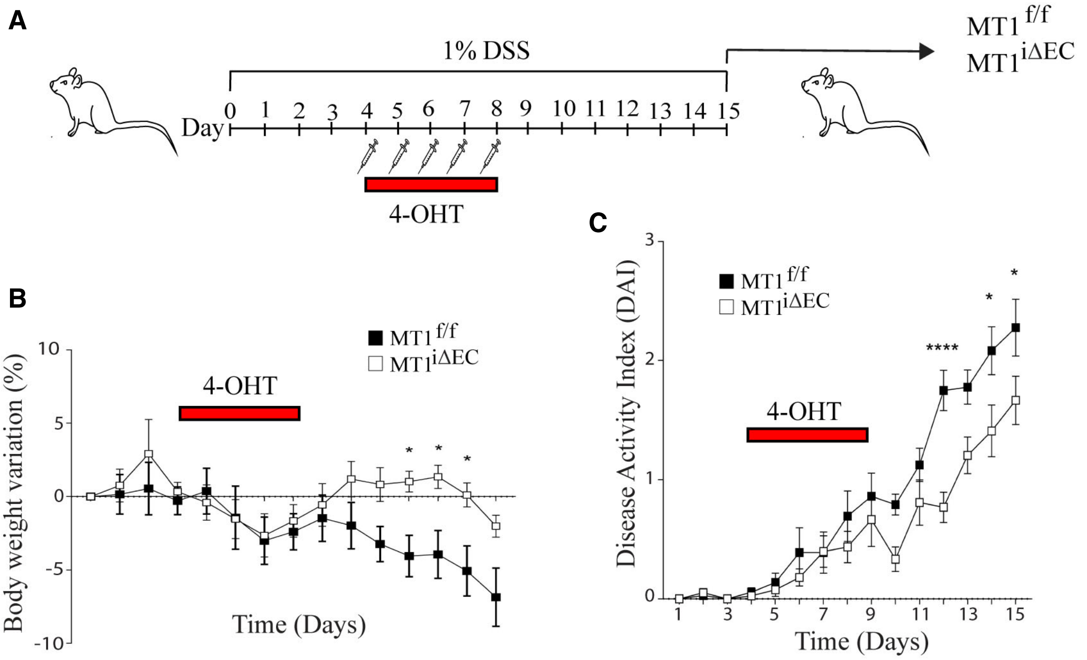

Figure EV3. Therapeutic deletion of MT1-MMP in endothelial cells hampers colitis progression.

A Experimental design for therapeutic deletion of MT1-MMP in endothelial cells during 1% DSS-induced colitis progression.

B, C Graphs show daily monitoring of body weight (% variation in B) and disease activity index (C) in $MT1^{f/f}$ and $MT1^{i\Delta EC}$ mice during the 15 days of 1% DSS treatment; n = 11–12 mice per genotype and condition. Data are shown as mean ± SEM and were tested by two-way ANOVA with Benjamini and Hochberg post-test; $*P < 0.05$, $****P < 0.0001$.

Data information: Please see Appendix Table S3 for exact P-values.

Source data are available online for this figure.

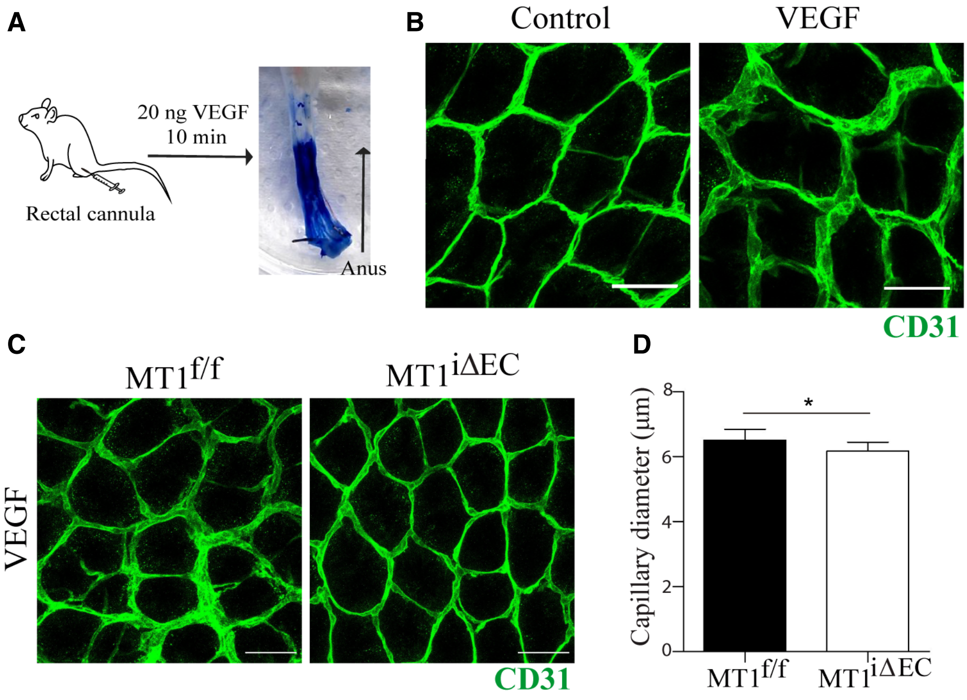

Figure EV4.

Figure EV4. Impaired VEGF-induced vasodilation of the colonic mucosa capillaries in the absence of endothelial MT1-MMP.

A Scheme depicting the method implemented for local administration of VEGF to the mouse distal colon via a rectal cannula and *in toto* macroscopic image of a colorimetric control (bromophenol blue).

B Representative images of staining for CD31 (green) in the mucosa vascular plexus from wild-type mice treated with saline or with 20 ng of VEGF in 50 μl via a rectal cannula as explained in (A). Scale bar, 25 μm.

C Representative images of staining for CD31 (green) in the mucosa vascular plexus from $MT1^{f/f}$ or $MT1^{i\Delta EC}$ mice after 10 min of rectal administration of 20 ng of VEGF. Scale bar, 25 μm.

D Quantification of capillary diameter in mice analyzed as in (C); n = 5–8 mice per genotype and condition. Data are shown as mean ± SEM and were tested by unpaired t-test; $^*P < 0.05$.

Data information: Please see Appendix Table S3 for exact P-values.
Source data are available online for this figure.

Figure EV5. Identification of TSP1 cleavage sites for MT1-MMP.

A Western blot of TSP1 expression (developed with an antibody against an epitope nearby the N-terminus; Lee *et al*, 2006) in lysates from siCtrl and siMT1-silenced HUVEC. MT1-MMP and tubulin are included as silencing and loading controls, respectively. The arrowhead marks full-length TSP1 and the asterisk the N-terminal TSP1 fragment.

B Western blot of *in vitro* digested TSP1 (developed with the same antibody as in A) incubated with increasing amounts of recombinant human MT1-MMP catalytic domain. rhMT1-MMP catalytic domain is also included. The arrowhead marks full-length TSP1 and the asterisk the N-terminal TSP1 fragment generated by MT1-MMP cleavage.

C *In silico* model of the membrane-anchored MT1-MMP dimer (blue/orange) and TSP1 type 1 repeat domains 2 and 3 (green). Yellow marks the catalytic pocket in the MT1-MMP protease, and red indicates the two selected cleavage sites in TSP1.

D Scheme depicting the TSP1 domain structure with the binding sequences to CD36, CD47, and αvβ3 integrin, as well as the positions of the identified cleavage sites for MT1-MMP.

E DAF-FM mean fluorescence intensity (MFI) in HUVEC expressing MT1-MMP siRNA and left untreated or treated with 200 ng of full-length TSP1 or the E123CaG-1 fragment; n = 128–184 cells analyzed per condition in two independent experiments. Data are shown as individual cell values and mean ± SEM and were tested by the Kruskal–Wallis test; $^*P < 0.05$, $^{***}P < 0.001$.

F Representative maximum-intensity projection images of CD31 staining (green) in whole-mount colon mucosal plexus of $MT1^{f/f}$ mice injected intraperitoneally with 1 μg full-length TSP1 or the E123CaG-1 fragment at day 0 and day 2 during 1% DSS treatment for 3 days. Scale bar, 40 μm. Arrows, arrowheads, and asterisks indicate duplications, loops, and pillars, respectively; n = 6 and 7 mice per condition in two independent experiments.

G Quantification of IA events in mice treated as in (F). Data are shown as mean ± SEM and were tested by t-test; $^*P < 0.05$.

Data information: Please see Appendix Table S1 for exact P-values.
Source data are available online for this figure.

© 2019 The Authors

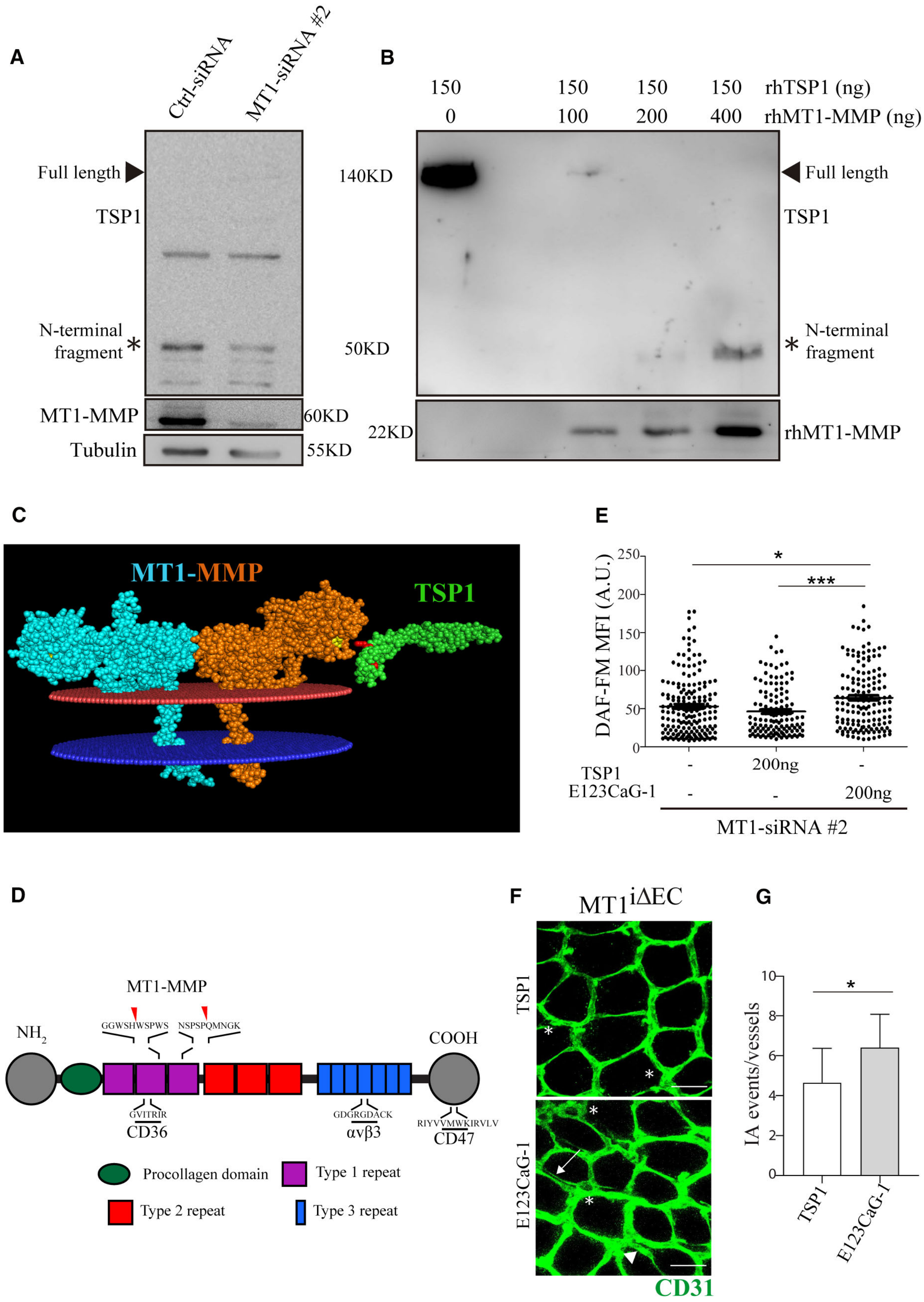
A
Ctrl-siRNA
MT1-siRNA #2
Full length
TSP1
N-terminal fragment
*
MT1-MMP
Tubulin
140KD
50KD
60KD
55KD
B
150 150 150 150 rhTSP1 (ng)
0 100 200 400 rhMT1-MMP (ng)
Full length
TSP1
N-terminal fragment
22KD
rhMT1-MMP
C
MT1-MMP
TSP1
E
DAF-FM MFI (A.U.)
250
200
150
100
50
0
*
***
TSP1 - 200ng -
E123CaG-1 - - 200ng
MT1-siRNA #2
D
MT1-MMP
GGWSHWSPWS
NSPSPQMNGK
NH2
COOH
GVITRIR
CD36
GDGRGDACK
αvβ3
RIYVVMWKIRVLV
CD47
Procollagen domain
Type 1 repeat
Type 2 repeat
Type 3 repeat
F
MT1iΔEC
TSP1
E123CaG-1
CD31
G
IA events/vessels
10
8
6
4
2
0
*
TSP1
E123CaG-1

Figure EV5.
