# Supplementary material for: Endothelial MT1‐MMP targeting limits intussusceptive angiogenesis and colitis via TSP1/nitric oxide axis
Source: EMBO Mol Med. 2019 Dec 3;12(2):e10862. doi: 10.15252/emmm.201910862 (PMC7005619; doi:10.15252/emmm.201910862)
Supplement: Supplementary file 5 — Source Data for Expanded View [file EMMM-12-e10862-s013.zip › Expanded_View_source_data/Source_Data_of_EV5.pdf]

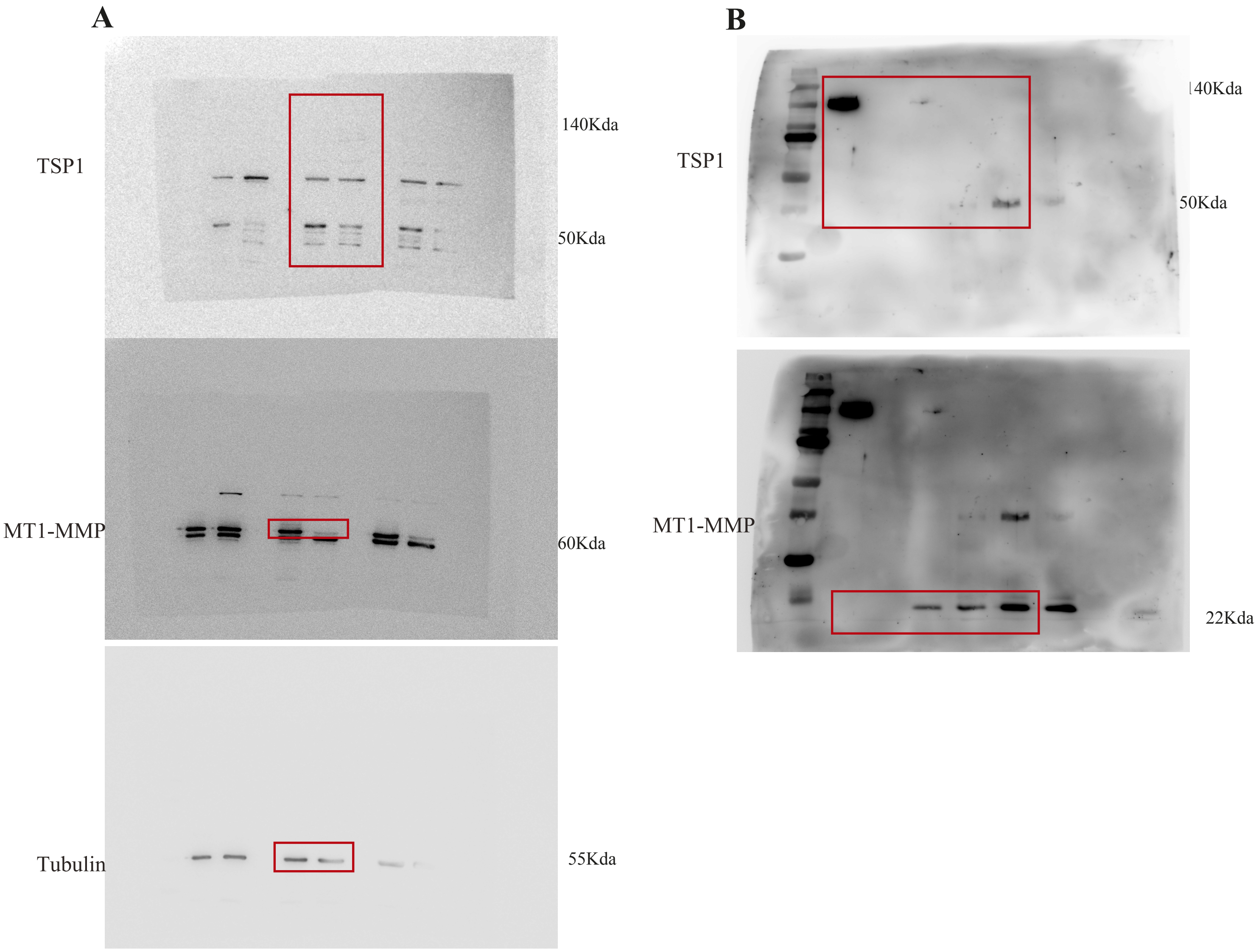

Source Data of EV5. Uncropped western blots used for EV5 pannels A and B. Red boxes indicate the bands that appear in the figure.
