# Supplementary material for: Endothelial MT1‐MMP targeting limits intussusceptive angiogenesis and colitis via TSP1/nitric oxide axis
Source: EMBO Mol Med. 2019 Dec 3;12(2):e10862. doi: 10.15252/emmm.201910862 (PMC7005619; doi:10.15252/emmm.201910862)
Supplement: Supplementary file 11 — Source Data for Figure 5 [file EMMM-12-e10862-s009.zip › Figure5/Source_Data_of_Figure_5.pdf]

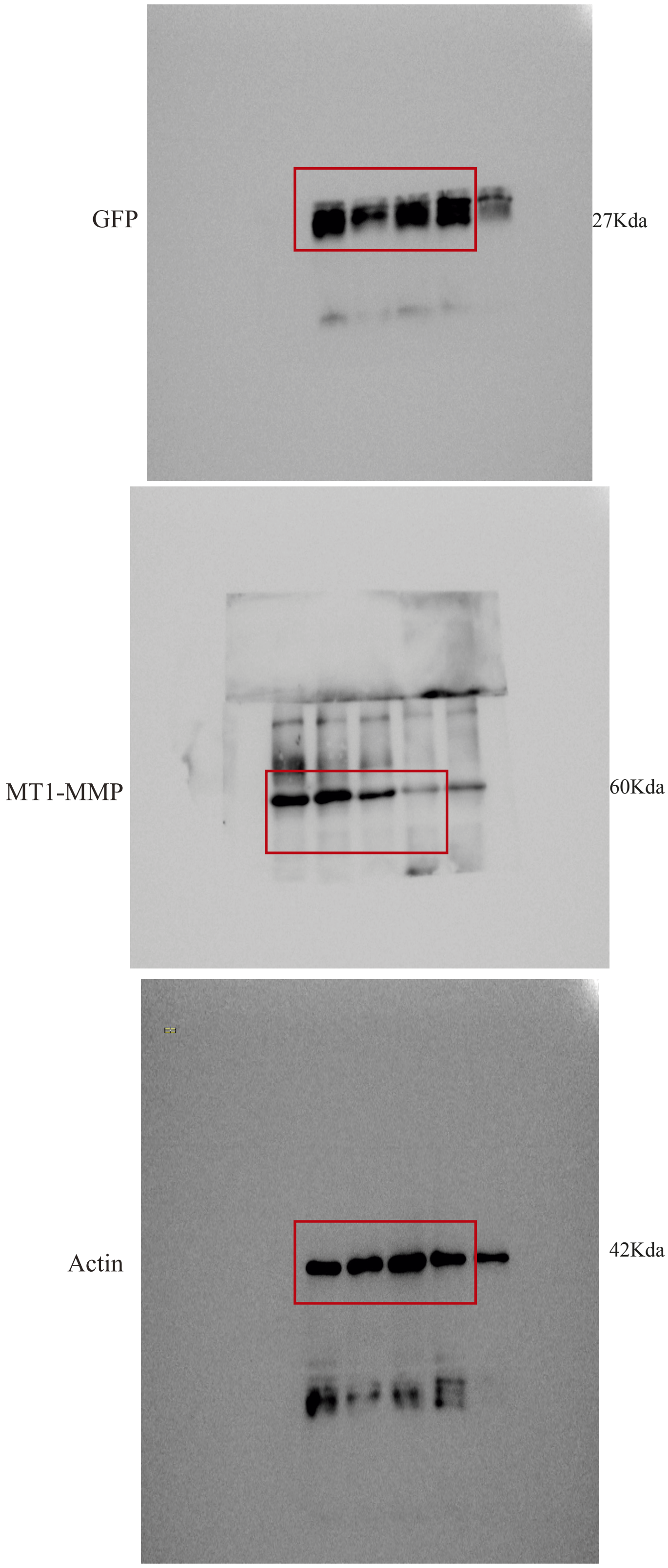

Source Data of Figure 5. Uncropped western blots used for Main Figure 5C. Red boxes indicate the bands that appear in the figure.
